# Lumbar degenerative disease after oblique lateral interbody fusion: sagittal spinopelvic alignment and its impact on low back pain

**DOI:** 10.1186/s13018-020-01837-w

**Published:** 2020-08-14

**Authors:** Jia Li, Di Zhang, Yong Shen, Xiangbei Qi

**Affiliations:** 1 Department of Orthopaedic Surgery, The Third Hospital of Hebei Medical University, Shijiazhuang, 050051 People’s Republic of China; 2 The Key Laboratory of Orthopedic Biomechanics of Hebei Province, The Third Hospital of Hebei Medical University, Shijiazhuang, 050051 People’s Republic of China

**Keywords:** Lumbar degenerative disease, Oblique lateral interbody fusion, Sagittal spinopelvic alignment, Low back pain

## Abstract

**Background:**

We determined the incidence and risk factors of low back pain (LBP) in patients with lumbar degenerative disease after single-level oblique lateral interbody fusion (OLIF).

**Methods:**

We retrospectively reviewed 120 lumbar degenerative disease patients who underwent single-level OLIF. We compared preoperative and postoperative radiographic parameters, including segmental lordosis (SL), lumbar lordosis (LL), disk height (DH), pelvic incidence (PI), pelvic tilt (PT), sacral slope (SS), thoracic kyphosis (TK), and C7-sagittal vertical axis (SVA). Clinical outcomes were evaluated using the Oswestry Disability Index (ODI) scores and visual analog scale (VAS) scores for back and leg pain. All patients were followed up for at least 2 years.

**Results:**

Thirty-eight patients had postoperative LBP (VAS score for back pain ≥3; LBP group); the remaining 82 patients were in the non-LBP group. Age (*P* = 0.082), gender (*P* = 0.425), body mass index (*P* = 0.138), diagnosis (degenerative spondylolisthesis vs. lumbar spinal stenosis; *P* = 0.529), surgical level (*P* = 0.651), blood loss (*P* = 0.889), and operative time (*P* = 0.731) did not differ between the groups. In both groups, the ODI and VAS scores for back pain and leg pain significantly improved at the final follow-up compared with the preoperative scores (*P* = 0.003). Except for the VAS score for back pain (*P* = 0.000), none of the scores significantly differed between the two groups at the final follow-up (*P* > 0.05). In the non-LBP group, LL, SL, DH, TK, and SS significantly improved, while PT and C7-SVA significantly decreased at the final follow-up as compared with the preoperative values. In both groups, DH significantly improved postoperatively, with no significant between-group difference (*P* = 0.325). At the final follow-up, LL, PI-LL mismatch, PT, and C7-SVA showed significantly greater improvement in the non-LBP group than in the LBP group (*P* < 0.05). Multivariate analysis identified PT, PI-LL mismatch, and C7-SVA as significant risk factors for LBP after OLIF.

**Conclusion:**

OLIF for single-level lumbar degenerative disease had satisfactory clinical outcomes. PT, PI-LL mismatch, and C7-SVA were significant risk factors for postoperative LBP. Patients with appropriately decreased PT, improved C7-SVA, and PI-LL match experienced less LBP.

## Background

Lumbar degenerative disease is characterized by back pain, radiculopathy, and neurogenic claudication. The most common types of lumbar degenerative disease are degenerative spondylolisthesis and lumbar spinal stenosis. Spinal sagittal imbalance is believed to be crucial for the development of lumbar degenerative disease. Sagittal spinopelvic parameters are influenced by factors such as age, sex, body mass index, and pelvic incidence (PI). Studies have demonstrated that PI plays a critical role in the overall alignment of the spine, and influences other sagittal spinal parameters, specifically, lumbar lordosis (LL) and thoracic kyphosis (TK) [1–3]. Indeed, sagittal spinopelvic alignment has become increasingly important for investigating preoperative planning and surgical outcomes in patients with lumbar degenerative diseases [4–6]. Failure to account for sagittal spinopelvic alignment might increase the risk of spinal misalignment and lead to poor clinical outcomes. Achieving an ideal spinopelvic alignment is recommended for optimal postoperative clinical outcomes [7–9]. Therefore, evaluations of spinal sagittal balance frequently inform surgical decision-making in lumbar degenerative disease [10–12].

Various lumbar interbody fusion techniques have been developed for the management of lumbar degenerative disease and are thought to be superior to conservative treatment. Fusion techniques confer several theoretical advantages, such as restoration of the disc height (DH), correction of spinal sagittal balance, and decompression of the neural foramina [13–15]. The reduction of LL is one of the causes of unsatisfactory clinical results after lumbar fusion, especially in patients with chronic persistent back pain.

Oblique lumbar interbody fusion (OLIF) is an emerging minimally invasive fusion technique that is increasingly being used for the treatment of lumbar degenerative diseases. The advantage of this minimally invasive technique is that injury to the paraspinal muscles, psoas muscle, and lumbar plexus can be avoided, as during OLIF, the intervertebral space is reached directly via a retroperitoneal channel [16–18]. Furthermore, OLIF can help restore DH through the use of larger cages as well as correct sagittal and coronal alignment, which indirectly decompresses the spinal canal. Despite these advantages, some patients who undergo OLIF continue to complain of residual low back pain (LBP) after the surgery. Sagittal spinal misalignment has been shown to be a risk factor for LBP after fusion surgery [19–21]. However, to our knowledge, no study has evaluated the factors influencing LBP after OLIF. We hypothesized that restoration of the sagittal spinopelvic alignment was beneficial to relieve LBP. To validate this hypothesis, we designed the present retrospective study, which aimed to determine the incidence of and risk factors for LBP after OLIF in patients with lumbar degenerative disease.

## Methods

### Study design and ethics statement

This study retrospectively reviewed the data of patients who underwent single-level OLIF surgery in our hospital between January 2015 and December 2017. The surgery was performed by the same team of surgeons in all patients. The inclusion criteria were as follows: patients who were diagnosed with symptomatic degenerative spondylolisthesis and/or lumbar spinal stenosis that could not be effectively managed using a conservative treatment for 3 months. Patients with isthmic spondylolisthesis, scoliosis, inflammatory spine disease, a history of lumbar or abdominal surgery, multi-level degenerative disease of the lumbar spine, trauma, malignancy, and infection were excluded from this retrospective study. The ethics committee of the Third Hospital of Hebei Medical University approved this study. Patient consent was not required for a review of medical records, as all data were de-identified. All protocols were conducted in accordance with the research principles set forth in the Declaration of Helsinki.

### OLIF procedure

All procedures were completed by the same surgical team. The patient was positioned in the right lateral decubitus position on the operating table. The intervertebral disc was approached with a blunt probe. To protect the posterior muscles and lumbar plexus, blunt dissection was performed through the plane between the retroperitoneal fat and the psoas muscle in the retroperitoneal space to access the lumbar spine. Discectomy was performed through this access portal. After opening the annulus fibrosus, the intervertebral disc and cartilage endplate were removed. A cage loaded with allogeneic demineralized bone matrix mixed with cancellous bone was inserted into the intervertebral space under intraoperative C-arm fluoroscopic guidance.

### Clinical measurements

Clinical and radiographic data collected preoperatively and at the final follow-up were analyzed in this study. For each patient, the following data were collected: age, gender, body mass index, diagnosis (degenerative spondylolisthesis or lumbar spinal stenosis), surgical level, operative time, and blood loss. The clinical measurements included the following: Oswestry Disability Index (ODI) questionnaires were administered for functional evaluation, and the visual analog scale (VAS) was used to assess back pain and leg pain.

**Fig. 1:**
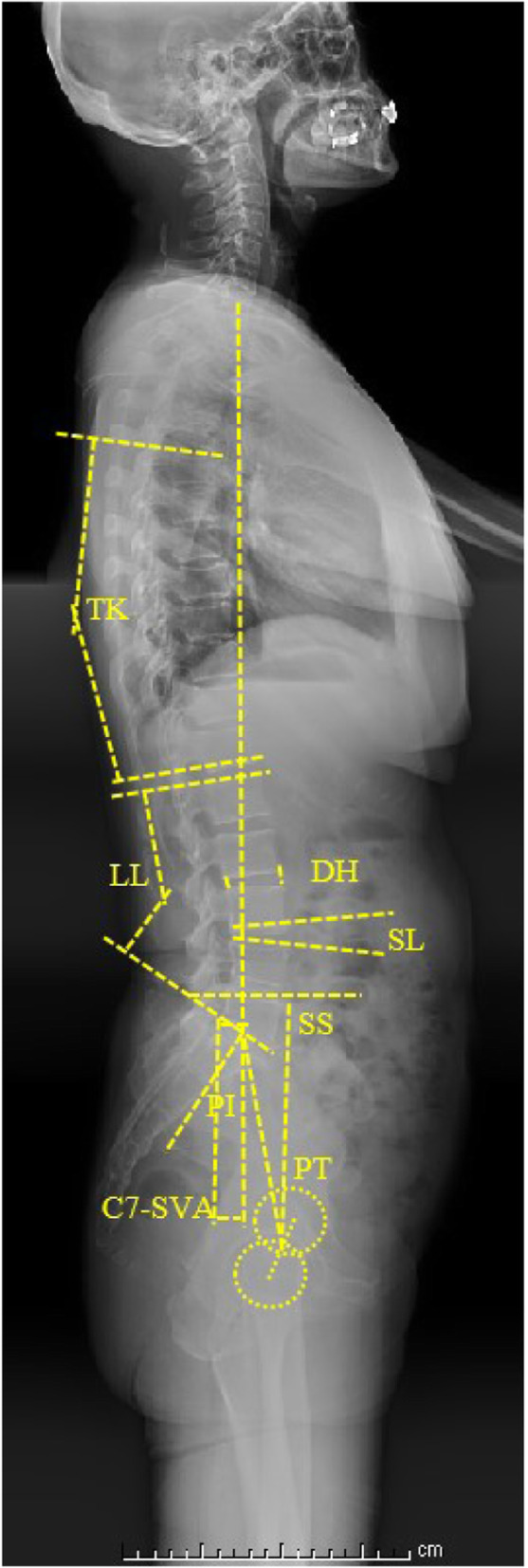
LL: the angle between the upper end plates of L1 and S1; SL: the angle between the lower endplate of the vertebra above the surgical level and the upper endplate of the vertebra below the surgical level; TK: the angle between the upper endplate of T5 and the lower endplate of T12; C7-SVA: the distance between the C7 plumb line and the posterosuperior border of S1; PI: the angle between the vertical line of the sacral endplate and the line connecting the midpoint of the sacral endplate to the midpoint of a line connected the centers of the femoral heads; PT: the angle between the line connecting the midpoint of the sacral endplate and the midpoint of the bilateral femoral head center and the C7 plumb line; SS: the angle between the horizontal plane and the sacral plate; DH: an average value of the anterior disc height and posterior disc height

All patients underwent standard, full spine X-ray radiography in the standing position. The patients were requested to stand in a relaxed and comfortable position while gazing straight ahead horizontally with their knees and hips in extension. The sagittal spinopelvic alignment in the standing position differs from that in the supine position. Although the PI remains stable across different body positions, the other sagittal spinopelvic parameters significantly differ among the various positions used in daily life. Therefore, the standing position was used to evaluate spinal balance in this study. All radiographic parameters were measured by two experienced orthopedic surgeons, and the average of their measurements was used for the analysis. The radiographic measurements included the following: LL was measured as the angle between the upper end plates of L1 and S1; segmental lordosis (SL) was measured as the angle between the lower endplate of the vertebra above the surgical level and the upper endplate of the vertebra below the surgical level; TK was measured as the angle between the upper endplate of T5 and the lower endplate of T12; C7-sagittal vertical axis (SVA) was measured as the distance between the C7 plumb line and the posterosuperior border of S1; PI was measured as the angle between a vertical line perpendicular to the sacral endplate at its midpoint and a line connecting the midpoint of the sacral endplate and the midpoint of a line connecting the centers of the two femoral heads; pelvic tilt (PT) was measured as the angle between the line connecting the midpoint of the sacral endplate and the midpoint of the bilateral femoral head center and the C7 plumb line; sacral slope (SS) was measured as the angle between the horizontal plane and the sacral plate; and DH was measured as the average value of the anterior and posterior DH (Fig. 1). The interobserver and intraobserver reliability of the preoperative values and the values at the final follow-up were >0.8, as estimated using interclass and intraclass correlation coefficients. Thus, the measurement methods used in this study were confirmed to be highly reliable.

### Statistical analysis

Statistical analyses were performed using SPSS software (version 22.0, Chicago, IL, USA). In all the analyses, a *P* value of <0.05 was considered to indicate a statistically significant difference. Differences between the preoperative measurements and the final follow-up measurements were analyzed using the paired-sample *t* test. The independent *t* test or chi-squared test was used to identify significant differences between groups. Multivariate logistic regression analysis was used to determine the risk factors related to LBP after OLIF. The results were presented as mean ± standard deviation.

## Results

### General information

The demographic data of the patients are summarized in Table 1. A total of 120 patients were included in this study. The mean follow-up period was 28.3 months (range 26–32 months). Patients who complained of LBP after OLIP were assigned to the LBP group (*n* = 38; VAS score for back pain ≥3), while patients without postoperative LBP were assigned to the non-LBP group (*n* = 82; VAS score for back pain <3). There were no significant differences between the LBP and non-LBP groups in terms of age (*P* = 0.082), gender (*P* = 0.425), body mass index (*P* = 0.138), diagnosis (degenerative spondylolisthesis vs. lumbar spinal stenosis; *P* = 0.529), surgical level (*P* = 0.651), amount of blood loss (*P* = 0.889), and operative time (*P* = 0.731). None of the patients required additional surgery on the surgical level or on adjacent levels for recurrent symptoms. Two patients developed thigh numbness, and another two patients developed transient thigh flexion weakness after OLIP. In all four patients, the symptoms resolved spontaneously within 3 months after the operation. Six patients were found to have cage subsidence after the surgery, while one patient underwent second-stage posterior fixation.

**Table 1:** Demographic data of patients in the LBP group and non-LBP group

	LBP group	Non-LBP group	***P*** value
Age (years)	60.5 ± 9.3	57.8 ± 10.2	0.082
Gender (M/F)	13/25	36/46	0.425
BMI (kg/m^2^)	29.1 ± 3.8	27.12 ± 5.1	0.138
Diagnosis			0.529
Degenerative spondylolisthesis	28	55	
Lumbar spinal stenosis	10	27	
Surgical level			0.651
L3–4	8	22	
L4–5	30	60	
Blood loss (mL)	106.6 ± 11.1	105.8 ± 9.9	0.889
Operative time (min)	95.9 ± 15.2	97.96 ± 11.8	0.731

*LBP* low back pain, *BMI* body mass index

### Clinical outcomes

In both the LBP and non-LBP groups, the ODI score and VAS scores for back pain and leg pain significantly improved at the final follow-up as compared with the preoperative scores (*P* = 0.003). Furthermore, except for the VAS score for back pain (*P* = 0.000), none of the scores significantly differed between the two groups at the final follow-up (*P* > 0.05).

### Radiographic outcomes

Statistical analysis showed that in the non-LBP group, the LL, SL, DH, and SS were all significantly improved while the PT and C7-SVA were significantly decreased at the final follow-up as compared with their preoperative values. At the final follow-up, DH did not significantly differ between the LBP (12.8 ± 1.9 mm) and non-LBP groups (13.1 ± 0.9 mm; *P* = 0.325). In the LBP group, the SL, PI, and TK were 7.5° ± 3.5°, 48.6° ± 12.2°, and 26.3° ± 11.5°, respectively, at the final follow-up, and these values did not significantly differ from the corresponding values in the non-LBP group (*P* > 0.05). In contrast, the PT at the final follow-up was significantly lower in the non-LBP group than in the LBP group (15.1° ± 7.3° vs. 22.3° ± 10.8°; *P* = 0.000). Furthermore, the LL at the final follow-up was significantly higher in the non-LBP group than in the LBP group (42.2° ± 11.2° vs. 35.8° ± 8.7°; *P* = 0.027). The PI-LL mismatch showed significantly greater improvement in the non-LBP group than in the LBP group (*P* = 0.006). Although the C7-SVA significantly decreased after OLIP in both the LBP group (from 51.8 ± 38.9 mm to 46.1 ± 37.9 mm) and the non-LBP group (from 45.1 ± 37.9 mm to 18.0 ± 28.5 mm), this parameter was significantly lower in the non-LBP group than in the LBP group at the final follow-up (*P* = 0.000; Table 2).

**Table 2:** Comparison of preoperative and the final follow-up radiographic parameters between the LBP group and non-LBP group

	LBP group		Non-LBP group	
	Preoperative	Final follow-up	Preoperative	Final follow-up
LL	32.6 ± 13.9	35.8 ± 8.7*	36.8 ± 10.9	42.2 ± 11.2*^,^ **
SL	4.1 ± 2.5	7.5 ± 3.5*	4.9 ± 3.2	8.8 ± 2.9*
TK	25.6 ± 12.2	26.3 ± 11.5	20.5 ± 11.7	24.5 ± 10.3*
C7-SVA	51.8 ± 38.9	46.1 ± 37.9*	45.1 ± 37.9	18.0 ± 28.5*^,^ **
PI	48.6 ± 12.2	48.6 ± 12.2	46.1 ± 8.2	46.1 ± 8.2
PT	23.9 ± 11.3	22.3 ± 10.8	19.8 ± 7.5	15.1 ± 7.3*^,^ **
SS	25.1 ± 9.7	26.9 ± 6.9	27.2 ± 8.9	31.7 ± 6.9*^,^ **
DH	8.5 ± 3.2	12.8 ± 1.9*	8.3 ± 2.1	13.1 ± 0.9*
PI-LL mismatch	19.8 ± 8.9	16.5 ± 6.8	9.8 ± 5.9	4.5 ± 3.6*^,^ **

*Indicates a significant difference between the preoperative and final follow-up values

**Indicates a significant difference between the LBP and non-LBP groups

*LBP* low back pain, *LL* lumbar lordosis, *SL* segmental lordosis, *TK* thoracic kyphosis, *SVA* sagittal vertical axis, *PI* pelvic incidence, *PT* pelvic tilt, *SS* sacral slope, *DH* disc height

To compare the relative impact of these variables on the incidence of LBP, we performed multiple logistic regression analysis. Variables with a *P* value of <0.2 in the univariate analysis, namely, age, SS, PT, LL, PI-LL mismatch, and C7-SVA, were analyzed as dependent variables, using a forward stepwise method. This analysis identified PT, PI-LL mismatch, and C7-SVA as significant risk factors for LBP after OLIF (Table 3).

**Table 3:** Comparison of preoperative and the final follow-up of the visual analogue scale (VAS) and Oswestry Disability Index (ODI) scores between the LBP group and non-LBP group

	LBP group		Non-LBP group	
	Preoperative	Final follow-up	Preoperative	Final follow-up
VAS score for back pain	5.9 ± 2.4	3.5 ± 1.2*	5.2 ± 2.2	1.7 ± 1.1*^,^ **
VAS score for leg pain	5.1 ± 1.4	2.1 ± 1.5*	5.3 ± 1.6	1.5 ± 1.1*
ODI score	25.5 ± 7.4	12.0 ± 4.7*	25.1 ± 6.7	11.5 ± 3.3*

*Indicates a significant difference between the preoperative and final follow-up values

**Indicates a significant difference between the LBP and non-LBP groups

*LBP* low back pain

## Discussion

This study demonstrated that unadjusted pelvic retroversion (i.e., insufficiently decreased PT), leaning forward position of the body (i.e., insufficiently decreased C7-SVA), and PI-LL mismatch were independent risk factors for LBP after OLIF. An insufficient decrease in PT after OLIF implied that the pelvis continued to be retroverted and could not correct the forward-leaning position of the body and the PI-LL mismatch, which might be the cause of LBP.

It is well known that biomechanical changes caused by sagittal imbalance are involved in the pathogenesis of degenerative lumbar disease. The PI is an anatomic parameter that plays a fundamental role in sagittal balance and spinal degeneration. A higher PI indicates a higher SS and LL, which might lead to higher shear forces at the lumbosacral junction, and is one of the causes of spondylolisthesis [22–24]. Therefore, restoration of the sagittal spinopelvic parameters is essential for improving patients’ quality of life after surgery. It is particularly important to restore an adequate sagittal spinopelvic alignment when performing spinal fusion surgery. It has been hypothesized that sagittal malalignment is a risk factor strongly correlated with LBP in patients after surgery [25, 26]. Many studies have reported that increased SS and LL after posterior lumbar surgery may lead to better clinical outcomes and less LBP. Failure to achieve proper sagittal balance results in compensatory mechanisms such as decreased SL and LL, and increased PT, which have adverse effects on the back muscles and eventually lead to LBP [7, 24, 27]. Recently, Liow et al. reviewed 63 patients who underwent short-segment lumbar fusion surgery and found that patients with higher SS (SS ≥30°) experienced less LBP; in their opinion, increased LL and SS indicated better clinical outcomes and sagittal balance [28].

Recently, OLIF has become a popular method of treating lumbar degenerative disease, as it has the advantage of minimizing iatrogenic injury to the posterior vertebral structures when compared with posterior lumbar surgery. Theoretically, indirect neural decompression can be achieved by restoring the intervertebral height. Abbasi et al. performed 303 OLIF procedures on 568 levels and reported that OLIF was a safe and efficacious procedure for lumbar degenerative disease [29]. Lin et al. found that OLIF could achieve equivalent clinical and radiologic outcomes by indirect decompression, as compared with other posterior lumbar surgeries, while achieving better restoration of DH and causing less blood loss [30]. Chang et al. also obtained favorable clinical outcomes after OLIF for lumbar spinal stenosis [31]. Consistent with the above studies, we observed significant improvement in clinical outcomes after OLIF and a minimum follow-up of 2 years in both our study groups (non-LBP and LBP groups), which were comparably matched in terms of demographic data and clinical outcomes.

Although OLIF can effectively lead to indirect spinal canal decompression and increased SS, some patients experienced residual LBP after the surgery. The current study showed that in the non-LBP group, the SS at the last follow-up (31.7° ± 6.9°) had significantly improved compared with the preoperative value. In contrast, in the LBP group, the SS at the final follow-up (26.9° ± 6.9°) was significantly lower than the corresponding value in the non-LBP group. However, multiple logistic regression analysis showed that SS was not a risk factor for LBP after OLIF. It is known that increased PT indicates pelvic retroversion, which compensates for the sagittal spinal imbalance. A PT of <20° is recommended to correct the sagittal imbalance and relieve symptoms [24]. In this study, the PT at the final follow-up was 22.3° ± 10.8° and 15.1° ± 7.3° in the LBP and non-LBP groups, respectively, and these values significantly differed from the preoperative values (*P* = 0.000). These results suggest that the degree of the decrease in PT in the LBP group was not enough to compensate for the sagittal imbalance and was associated with residual back pain.

In addition, many research studies have reported that increased LL and SL are correlated with improved clinical outcomes [32–34]. Our results showed that SL was significantly improved after single-level OLIF in both groups. Although the SL in the non-LBP group was slightly higher than that in the LBP group, the difference was not statistically significant. However, the LL in the non-LBP group was significantly higher than that in the LBP group. This suggested that the impact of the interbody fusion was not enough to alter the overall spinal sagittal alignment, despite the placement of a large cage on both sides of the endplate and anterior to the vertebral body during OLIF.

The C7-SVA has been reported to be an important index of sagittal imbalance [3, 6, 7]. In our study, the C7-SVA had significantly decreased in both groups at the final follow-up. The change in C7-SVA was greater in the non-LBP group than in the LBP group. Additionally, a PI-LL mismatch of <10° was used to indicate whether sagittal reconstruction had been achieved in the non-LBP group. We found that OLIF could improve LL and correct PI-LL mismatch. Furthermore, the decreased C7-SVA was as evidenced by the adjustment of LL. Saadeh et al. reported that single-level lateral lumbar interbody fusion greatly improved regional lordosis, but global lordosis was not impacted by the single-level intervention [35]. Schwab et al. showed that postoperative PI-LL mismatch causes greater residual LBP and proposed that SVA, PT, and PI-LL mismatch were most closely related to poor clinical outcomes and LBP [36].

Although surgery improved DH, LL, SL, PI-LL mismatch, and C7-SVA, an ideal sagittal balance could not be achieved in the LBP group. OLIF could only partially restore sagittal balance by increasing the intervertebral height through the placement of a large interbody cage anteriorly within the wider distraction of the intervertebral space. On the one hand, deficient vertebral distraction is insufficient for spinal decompression and affects the correction of sagittal imbalance. On the other hand, excessive vertebral distraction necessitates the use of an overlarge interbody cage, which increases the risk of subsidence into the endplate, reduces fusion rates, and significantly increases mechanical stress on adjacent discs. Furthermore, the position of the interbody cage affected the recovery of the intervertebral height, which indirectly affected the restoration of LL and SL. Therefore, the placement of a larger intervertebral cage in the anterior- or middle-third of the spinal column would improve the sagittal spinopelvic alignment. However, with regard to the SL, each spinal level contributes a different and limited magnitude to the LL. Therefore, we considered that restoration of the intervertebral height by cage insertion might be insufficient to alter the mechanical dynamics of the spine.

To our knowledge, this is the first study to investigate the incidence of LBP and the impact of sagittal spinopelvic alignment on patients after OLIF. It is particularly important to identify and restore sagittal spinopelvic alignment when performing this procedure. However, this study has several limitations. First, this was a single-center, retrospective study with a small sample size and a relatively short follow-up period. Further studies with larger cohorts followed up for longer periods are needed. Second, in the current study, one patient underwent second-stage posterior fixation; most patients did not undergo posterior fixation; whether posterior fixation is required for all patients needs to be determined using a longer follow-up period. Third, although OLIF restored DH and corrected LL in patients with minor sagittal imbalance, it is unclear whether OLIF will result in similar corrections in patients with degenerative scoliosis or excessive imbalance. Furthermore, the ideal method for correcting sagittal spinopelvic alignment to maintain optimal postoperative sagittal balance is difficult to determine.

## Conclusion

The present study demonstrated that the clinical outcomes of single-level OLIF for the surgical treatment of lumbar degenerative disease were satisfactory after a minimum follow-up period of 2 years. PT, PI-LL mismatch, and C7-SVA were identified as significant risk factors for LBP after OLIF. Patients with appropriately decreased PT, improved C7-SVA, and improved PI-LL mismatch experienced less LBP. These findings provide some guidance for identifying and restoring sagittal spinopelvic alignment when performing this procedure.

## Data Availability

Not applicable.

## Abbreviations

DH: Disc height
OLIF: Oblique lumbar interbody fusion
LBP: Low back pain
ODI: Oswestry Disability Index
VAS: Visual analog scale
LL: Lumbar lordosis
SL: Segmental lordosis
TK: Thoracic kyphosis
SVA: Sagittal vertical axis
PI: Pelvic incidence
PT: Pelvic tilt
SS: Sacral slope
